# Photorefraction Simulates Well the Plasticity of Neural Synaptic Connections

**DOI:** 10.3390/biomimetics9040231

**Published:** 2024-04-13

**Authors:** Alessandro Bile, Hamed Tari, Riccardo Pepino, Arif Nabizada, Eugenio Fazio

**Affiliations:** Department of Fundamental and Applied Sciences for Engineering, Sapienza Università di Roma, Via Scarpa 16, 00161 Roma, Italy; hamed.tari@uniroma1.it (H.T.); riccardo.pepino@uniroma1.it (R.P.); arif.nabizada@uniroma1.it (A.N.); eugenio.fazio@uniroma1.it (E.F.)

**Keywords:** artificial intelligence, neuromorphic systems, neuroplasticity, neural networks, learning, photorefractive solitons, photonic hardware, all-optical systems

## Abstract

In recent years, the need for systems capable of achieving the dynamic learning and information storage efficiency of the biological brain has led to the emergence of neuromorphic research. In particular, neuromorphic optics was born with the idea of reproducing the functional and structural properties of the biological brain. In this context, solitonic neuromorphic research has demonstrated the ability to reproduce dynamic and plastic structures capable of learning and storing through conformational changes in the network. In this paper, we demonstrate that solitonic neural networks are capable of mimicking the functional behaviour of biological neural tissue, in terms of synaptic formation procedures and dynamic reinforcement.

## 1. Introduction

The human brain is a complex computer system, capable of collecting data, processing them and storing them [1]. Through this succession of operations, the brain learns, i.e., it knows from experience. Its architecture is perfectly optimized, as it is able to perform a large number of operations with minimal energy consumption [2]. This is possible because the brain exploits all the three spatial dimensions to distribute its functionality. In particular, it works using neurons, elementary units of the neural system, for both the calculation and storage of information, thus exercising a fundamental dual function of processor and memory. This means that no time and energy are required to retrieve information, process it and store it [3]; everything happens simultaneously in the same physical location in the brain, and, therefore, with minimal effort. In contrast, traditional computers exploit Von Neumann’s architecture, whereby all hardware elements, processors—memories—peripherals, are distinct and distant entities, physically separate, requiring a great deal of energy and time to communicate with each other. For these reasons, scientific research has investigated the characteristic geometries and mechanisms of brain functioning in order to replicate them in advanced computer systems. This approach is known as the “neuromorphic approach” and refers to the study of hardware that simulates the typical functioning of neurons and neural networks, i.e., based on distributed elementary units, capable of simultaneously processing and storing according to the calculation protocols typical of biological systems.

## 2. Biological Neurons and Neural Networks

**Figure 1:**
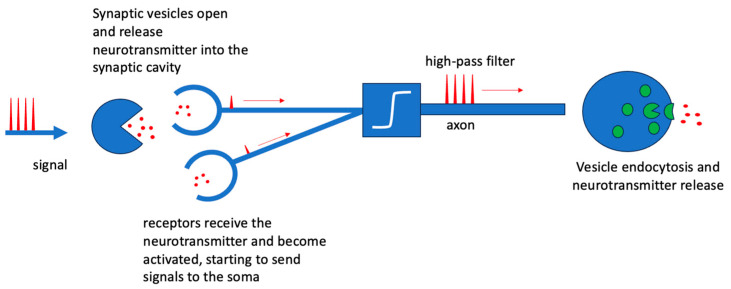
Diagram of synaptic activation upon the onset of a supra-threshold signal. The signal, propagating along the axon, causes the movement of vesicles that collect neurotransmitters and carry them towards the synaptic cavity where they are released by the endocytosis of the vesicle itself. New vesicles, inside which no neurotransmitters are present, are subsequently formed and move in the opposite direction towards the centre of the cell.

The fundamental units of computation in the nervous system are neurons [1]. A neuron typically receives information from previous units, processes it, and redistributes it to the subsequent neurons connected to it. The interconnections between neurons are called synapses. Not all synapses available to a neuron are active, only those that are functional to the specific signal processing. The set of active neurons and synapses constitutes the neural circuit. Neural networks formed in this way are dynamic networks, i.e., they can change in structure by activating and deactivating synaptic interconnections. A specific trajectory of active synapses in the neural network simultaneously identifies a reasoning and a memory; in fact, a reasoning activates a well-defined set of synaptic interconnections between neurons and their mapping that, maintained over time, constitutes the memory of the reasoning, and thus, of the concepts and notions associated with it. In the neural network, the “strength” of the synaptic connection is of great importance; a strong connection will remain for a long time while a weak connection will soon fade, just as, in other words, an important memory will remain for a long time while an unimportant memory will quickly fade. This complex dynamic of interconnection between activated neurons goes hand in hand with a relatively basic method of information processing; indeed, each neuron performs operations as simple as summing up the incoming signals and comparing them as an high-pass filter. Let us see how. The arrival of a signal releases neurotransmitters in the region between the two synaptic dendrites of successive neurons (synaptic cavity). These substances are able to activate and strengthen the interconnection. The strength of the synaptic interconnection, thus, depends largely on the number of neurotransmitters that are released into the synaptic cavity and the number of receptors that receive them. The receptors of the next neuron act as input for signal propagation in the new neuron. As the number of neurotransmitters and receptors increases, the ability to transmit signals improves, and thus, the strength of the synaptic connection will also last longer. The neurotransmitter release dynamics (exocytosis) of pre-synaptic [2] vesicles are triggered by the arrival of a signal as shown in Figure 1. The $N_{v}$ vesicles fuse with the cell membrane and release $N_{T\;}$ neurotransmitters into the synaptic cavity. After release, the vesicles reform, but remain devoid of neurotransmitters within $N_{v}^{0}$. Therefore, the rate of the release of free neurotransmitters is governed by Equation (1) as follows:

(1) $$\frac{\partial N_{v}^{0}}{\partial t}=\sigma SN_{v}-\gamma N_{v}^{0}N_{T}$$

where *S* corresponds to the input signal, *σ* is the neurotransmitter release efficiency as a function of the input signal, and *γ* is the probability of encounter between an empty vesicle and a neurotransmitter. The number of neurotransmitters present within the synaptic cavity is, therefore, governed by the dynamics of vesicle release; it may increase due to vesicle release and may decrease either by reabsorption in the reformed vesicles or by diffusion into the surrounding environment. Therefore, the rate equation of the density of neurotransmitters present in the synaptic cavity (and identifying the strength of the interconnection) will be similar to that of the vesicles with the addition of a diffusion term as follows:

(2) $$\frac{\partial N_{T}}{\partial t}=\frac{\partial N_{v}^{0}}{\partial t}-\vec{\nabla }\cdot \vec{J}$$

**Figure 2:**
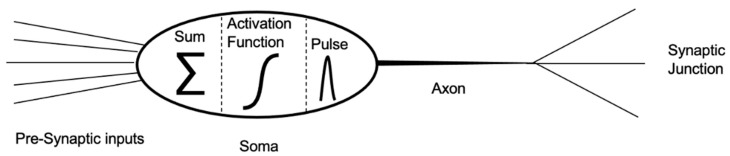
Schematic diagram of the functional structure of a neuron; the neuron collects presynaptic input data via the dendrites. Once in the soma, the signals are summed. If the resulting value exceeds a reference threshold, the soma transmits a signal, a long output channel, to the axon and distributes it to the synaptic junctions with the following neurons. If the sum of the signals in the soma does not exceed the reference threshold, the neuron does not transmit and is considered inhibited.

Neurotransmitters are, therefore, released from the pre-synaptic neuron when an electrical stimulus occurs. The more intense the stimulus, or the more frequently it occurs, the greater the release of neurotransmitters. The onset of a signal only stimulates the release of neurotransmitters if above a certain threshold. Let us see how signal processing occurs. Figure 2 shows the different functional districts of a neuron. The presynaptic input channels, called dendrites, receive information from the preceding neurons and transfer it to the soma, which is the processing centre. In the soma, the received signals are summed up and the result is compared to a threshold level described by a high-pass activation function; if the sum of the received signals exceeds the threshold, then the neuron activates and starts transmitting to the axon (spiking or firing operation). Otherwise, if the sum of the signals does not reach or exceed the threshold, then the neuron will be inhibited and will not transmit any further. This behaviour is typical of electronic high-pass filters; a signal above the threshold will pass while a signal below the threshold will be blocked [1,2]. Once in transmission, the neuron sends the signal along the axon that redistributes it, without changing its amplitude, to subsequent neurons via the outgoing synaptic junctions.

As it exits the axon, the signal transmitted from the soma is divided and distributed across the synaptic junctions to successive interconnected neurons. Each dendritic termination releases neurotransmitters according to the process described above, modulating the various synaptic connections. There are mainly two situations that create marked synaptic pathways, i.e., privileged pathways: (1) when a piece of information is repeated several times (the same specific pathway is retraced several times, increasing the number of neurotransmitters at each step, and thus, making the interconnections stronger and longer lasting), or (2) when the information is new, novel and very strong. The first case can be traced back to a process like school or academic study; by continuing to study and repeat, information is memorized and will remain mnemonic for a long time. The second case can be traced back to a sudden, violent, upsetting process, such as a shock, a traumatic event or a sudden, beautiful event. In this second case, neurotransmitters are released instantaneously into the various synaptic connections during propagation, producing a very strong and well-defined neural pathway without the need for subsequent reinforcement iterations. This is nature’s way of learning that a certain action may be harmful or reminds us of moments of extreme happiness. Similarly, for unused pathways, the number of neurotransmitters in the synaptic cavities slowly decreases, making the pathway less and less defined until it slowly dissolves (forget). However, signal propagation may be subject to loss. Neural channels are covered by myelin in order to limit the loss of information. Whether the signal propagates without leakage depends largely on the presence and amount of myelin around it [4]. Myelin is an insulating substance consisting mainly of lipids and proteins, which externally coats axons [2]. This coating may be simple, i.e., a single layer, or composed of several concentric layers, giving rise to a kind of sheath or sleeve. This sheath is not continuous but interrupted, forming many segments; in their presence, propagated signals travel faster as they jump from one segment to another. This “jump conduction” increases the speed of transmission as conduction is more efficient; furthermore, the presence of myelin, in addition to bringing nourishment to the neuron, mechanically protects it and, above all, isolates it by avoiding crosstalk between neighbouring neurons. In fact, in the absence of myelin, especially where neuronal networks are particularly dense, neurons could respond to the multiple surrounding signals carried by other neurons: just as an electric wire without an insulating coating would disperse the current without carrying it to its destination. Myelin promotes signal confinement by eliminating unwanted crosstalk between neighbouring neurons or axons. Therefore, the biological brain is a dynamic system, capable of changing its geometry, reorganizing the map of neural interconnections to learn new information or to forget old ones. This adaptive capacity of biological networks is the real challenge of neuromorphic hardware research.

## 3. Photonic Neuromorphics

Neuromorphic electronics is inspired by the functioning of the biological nervous system and seeks to develop circuits that can learn, adapt and process information. Unlike traditional electronic circuits, which are largely static and pre-programmed, neuromorphic devices must be able to adapt and learn from experience. This is made possible by artificial neural networks that simulate the way biological neurons communicate and form synaptic connections. Despite the extreme integrability of devices, neuromorphic electronics has failed to realize compact electronic neurons. It succeeded in realizing extended circuits with responses similar to those of single neurons. This brings topological limitations (especially for interconnections in dense and ultra-dense networks), together with the technological limitations typical of conventional electronic circuits, such as limited bandwidth, high power consumption, high heat generation and poor electromagnetic compatibility. Compared to electronics, photonics shows considerable advantages [3]; it has a far greater bandwidth, limited energy consumption [5], may not require physical interconnections between circuit elements, and favours parallel computing in dense or ultra-dense networks. In addition, light is able to exploit the nonlinearities of the host material more effectively. This last factor is of utmost importance to realize complex devices or systems capable of self-modifying to learn information. Thus, research has slowly moved towards optics, first with optoelectronic neuromorphic circuits and then towards fully optical systems [6]. So far, the main attempts at neuromorphic optics have followed two paradigms:

(1) the realization of a single neuron;
(2) the realization of active connections between neurons.

The first paradigm attempts to reproduce the main functions of biological neurons in integrated photonic systems. It exploits excitability, i.e., the ability to reproduce and transmit self-consistent signals from even weak inputs according to a high-pass filter-like threshold process [6,7,8,9,10,11]. Sub-threshold or amplifier laser systems [6,7] are exploited to reproduce this behaviour, including graphene-impregnated semiconductor lasers [8], photonic crystals [9], vertical cavity lasers [9] or resonant tunnelling photodetectors [11,12]. These systems have major limitations due to the lack of nonlinearity that can be used to store information. Therefore, they are also unable to carry out learning at the same time and are, therefore, unable to adapt to the type of information received. To resolve this limitation, the second paradigm intervenes, focusing not on individual devices but on their interconnections. In fact, the second neuromorphic paradigm is based on the possibility of opening and closing interconnections, and thus, realizing specific trajectories in the neuronal network, recording all signal passages. By doing so, the entire map of the activated synapses constitutes the stored information, simple or complex, which can be reinforced or possibly deleted in order to forget. The second paradigm is based on neural plasticity, i.e., the ability of a nervous system to modify itself, to adapt to information, storing it in the form of specific trajectories in the processing network [13,14]. To achieve a memory-network, it is, therefore, necessary to be able to open and close, as well as reinforce, each connection. The strength of the interconnection is, in fact, the necessary tool to realize and optimize a mapping. In O/E/O neuromorphic devices, this is usually implemented electronically, as demonstrated by the photoreceptor–modulator neuron model for Mach Zehnder Interferometer networks [15] and the electronic superconducting significant pathway [16]. Fully optical implementations of weighted interconnections, e.g., by exploiting chalcogenic phase change materials (PCMs) [17,18], are also already present in the literature. In short, the crystalline state, and thus, the optical properties of these materials depend on the different received light signals. Synapses consist of optical waveguides and the reinforcement process is performed by PCM cells that can absorb or transmit light in accordance with their crystalline state, which, in turn, depends on the received light. Thus, when the PCM is in the amorphous state, light absorption is low and a strong connection occurs, whereas if the PCM is in the crystalline state, most of the light is absorbed, making the connection weak. PCM materials are a good solution for superimposing memory and processing units but have important limitations; they can only assume two states and have no memory of the previous state. Recently, photorefraction has been effectively employed to realize active solitonic interconnections [19], i.e., capable of establishing, reinforcing or possibly even erasing themselves. Photorefraction had already been employed in the past to store even complex information in the form of holographic patterns. However, the great novelty of photorefraction lies in the possibility of realizing innovative interconnections in the form of solitonic waveguides. By exploiting photorefractive nonlinearity, spatial solitons can be formed. Thus, laser beams do not diffract because they have changed the refractive index of the host material, so that induced waveguides are written. The writing process is also nonlinear, allowing each individual channel to be reinforced, thus making the index contrast of the waveguide ‘stronger’. Thus, photorefraction is the ideal tool to simulate the plasticity of biological neural systems [20].

## 4. Photorefraction as a Basis for Optical Neural Networks

Photorefractive nonlinearity is based on the electro-optical effect, i.e., the change in the refractive index of a material subjected to a static electric field. A photorefractive material is typically a semiconductor capable of absorbing light by transitions from trap states (gift states) to the conduction band. Thus, the absorption of light will generate two species of carriers: electrons free to move in the conduction band and holes located in trap states. This behaviour is usually described by the following regime equations for ionized $N_{D}^{+}$ donors and $N_{e}$ electrons:

(3) $$\frac{\partial N_{D}^{+}}{\partial t}=\sigma FN_{D}-\gamma N_{D}^{+}N_{e}$$

(4) $$\frac{\partial N_{e}}{\partial t}=\frac{\partial N_{D}^{+}}{\partial t}-\vec{\nabla }\cdot \vec{J}$$

In Equation (3), *F* is the photon flux, *σ* the absorption cross-section, *γ* the decay probability while *J*, in Equation (4), is the current density, due to both conduction and electrical scattering. The charges thus generated, both free electrons and localized gaps, generate a local electric field capable of changing the refractive index of the photo-refractive material due to the electro-optical effect. This change in refractive index will be local because the static charge distributions are local and, therefore, the photoinduced electric field will be local [21,22]. By focusing a Gaussian laser beam in a photorefractive material, the bell-shaped intensity distribution will induce a micro-lens-shaped refractive index change that can refocus the light. By extending this behaviour throughout the material, it is possible to obtain narrow channels of higher refractive index that act as waveguides, within which light can be trapped. This describes the formation of a spatial soliton, i.e., a self-confined laser beam within the refractive index variation induced by it. But this variation can also be effective at other wavelengths “being a real variation”, which can exploit the soliton channels as waveguides, within which it can travel confined. This process is quite similar to the behaviour of biological synapses. In fact, local index variation depends on the gradient of electrical charges and ionized donors that assume the functional role that neurotransmitters play in biological neural networks (BNNs): regulating synaptic intensity. When the strength of a biological synapse changes, the neural system begins to learn. Repetition of the same pattern of signal input results in synaptic strengthening, which is synonymous with information storage [12]. Therefore, a structural change in the connection (strengthening or weakening) can mean memorization or forgetting. Similarly, solitonic interconnections strengthen or weaken depending on the intensity of the information flowing through them and the number of times the information is replayed for learning. In summary, biological and solitonic networks learn by modifying the network of connections according to incoming information. A synapse is strengthened mainly by two factors:

- the intensity of incoming signals;
- the frequency with which the signals occur in a specific pathway.

Both processes lead to the release of a higher number of neurotransmitters into the selected synaptic cavity, ensuring a higher binding intensity.

**Figure 3:**
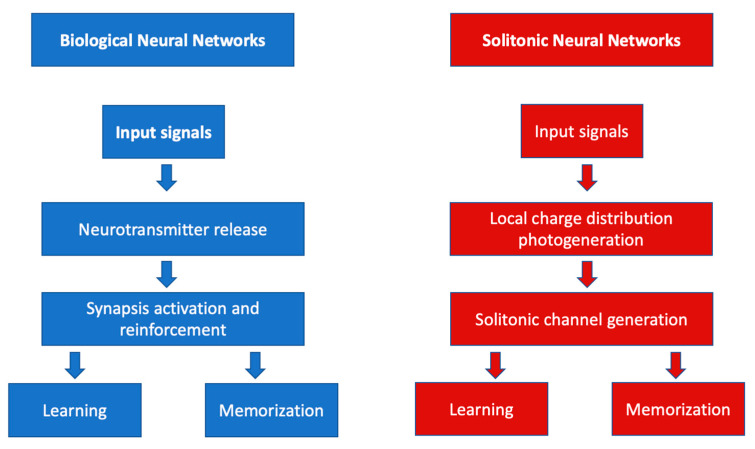
Diagram of the functioning of learning and memorising processes of a biological neural system (**left**) and a solitonic neural system (**right**).

Figure 3 shows the functional parallelism between BNN and SNN.

**Figure 4:**
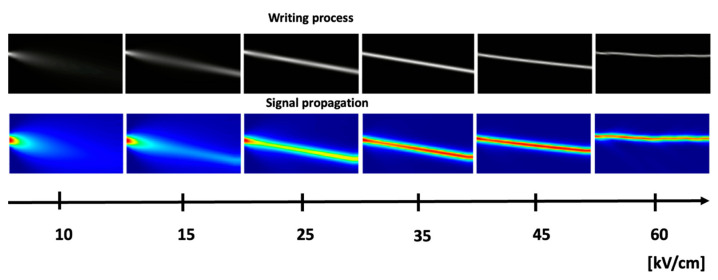
From left to right: the evolution of the formation of a solitonic waveguide (first line) and the propagation of a signal in it (second line) as a function of the electric polarisation field. As the E_bias_ increases, the confinement of light increases, and signal propagation is more focused. Blue represents low intensity while red is the maximum intensity. Above 50 kV/cm, a pulsating phenomenon occurs, and the waveguide begins to lose some of its signal.

In the biological neural networks, input signals cause the release of neurotransmitters that induce the activation and reinforcement of synapses responsible for the evolution of biological synapses. In the photonic–solitonic case, the input signals are responsible for the photoexcitation of electrical charges. The photoproduction of ionized donors and free electrons depends on the intensity of the incoming light signal just as the number of neurotransmitters released depends on the intensity of the stimuli received and the frequency with which they occur. A higher density of neurotransmitters is synonymous with synaptic weight enhancement while higher densities of ionized donors and free electrons induce a higher refractive index change and, consequently, a greater ability of the waveguide to confine and transport information. Conversely, if the intensity of the electrical stimulus was low or no further stimulus was forthcoming, the concentration of the neurotransmitters would be low or even zero and the synaptic weight would weaken. If no light is injected into the crystal, the photo-ionization process is not initiated and no change in the refractive index is triggered. Thus, in the biological case, thanks to neurotransmitters, electrical signals can pass from one neuron to another, i.e., a synaptic connection can be opened. In the solitonic case, thanks to ionised donors, a solitonic waveguide can be formed and signals can pass from one point of the nonlinear crystal to another. In the biological case, the strength and speed of signal propagation depends on the number of neurotransmitters released; in the solitonic case, the strength of the solitonic propagation, i.e., the ability to transfer information without loss and scattering, depends on the index contrast of the guide, which, in turn, depends on the number of ionised donors influencing the local electric field. As can be seen, there is a direct correlation between the behaviour of biological neurotransmitters and the behaviour of ionised donors. The similarities between the biological and solitonic systems are also evident with regard to the properties of signal transmission. In BNNs, the efficiency of signal conduction depends on the amount of myelin present [23,24,25]. Its main effect is to ensure focused and loss-limited signal conduction. This dependence is very similar to that between light propagation in the solitonic regime and the static electric field of polarisation. Higher values of this field correspond to a higher self-confinement of the light, and thus, to a more significant focusing of the signal. Figure 4 shows numerical simulations of the formation of the solitonic channel (first line in black and white) and the propagation of the signal (second line in colour) within it. For bias values of less than 15 kV/cm, diffractive behaviour prevails over self-focusing; therefore, the input signal is diffracted over the entire crystal (second line). Increasing the value of the bias electric field results in a more pronounced confinement, i.e., an efficient solitonic waveguide, which causes signals to propagate without diffraction. If the Ebias exceeds 50 kV/cm, however, instability begins to occur; the waveguide begins to pulsate and can no longer carry the signal, so losses occur.

**Figure 5:**
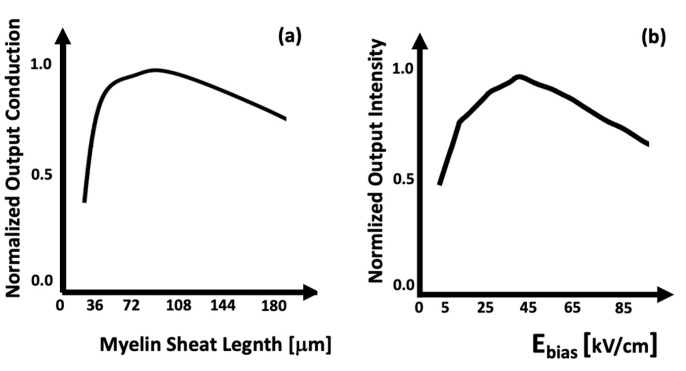
In (**a**), the trend of signal conduction as a function of myelin length. In (**b**), the trend of output signal strength as a function of the E_bias_ polarisation field.

The effect of myelin on biological signal conduction is compared with the effect of the polarization electric field on the propagation of optical information in Figure 5. Figure 5b shows the integral of the output signal as a function of the polarization field value. As E_bias_ increases, the intensity also increases up to a threshold value [26]. After this value, the channel instability increases; the signal is no longer fully contained and is lost along the path.

## 5. Conclusions

In conclusion, this manuscript demonstrates that optical photorefraction simulates the plastic behaviour of biological neural systems. Indeed, intelligent optical systems realised through spatial solitons are able to change their structure over time according to the signals they receive, just as biological units do. In the case of solitonic structures, the density of charge carriers plays a key role in the development of synaptic connections, similar to that played by the density of biological neurotransmitters. Just as the strength of a biological synapse depends on the concentration of neurotransmitters in the synaptic cavity, in the same way, the formation of a solitonic channel depends on the concentration of photo-ionized donors. Both of these quantities are driven by stimuli external to the system. Furthermore, the bias electric field, which underlies the photorefractive process, acts in a similar way to biological myelin; it can improve signal conduction, favouring the focusing of light and reducing diffractive leakage phenomena. Nowadays, there are many ways to obtain biological perception [27,28,29,30]. The great advantage of soliton technology over previous implementations lies in the possibility of making self-assembling and self-optimizing devices. In addition, since these is hardware that works completely in the optical domain, it is easily interfaced with plasmonic technology, which has been maturing important achievements just in recent years [31]. In this work, we have shown for the first time that, by exploiting the plastic behaviour of photorefraction, it is possible to create intelligent systems whose properties are similar to the mechanisms through which biological neurons are connected. Therefore, it is possible to speak of a solitonic neural tissue.

## Data Availability

The research data are generated by the direct application of the model and of its associated equations.
